# Cardiac MRI assessment of anthracycline-induced cardiotoxicity

**DOI:** 10.3389/fcvm.2022.903719

**Published:** 2022-09-27

**Authors:** Leila Mabudian, Jennifer H. Jordan, Wendy Bottinor, W. Gregory Hundley

**Affiliations:** ^1^Division of Cardiology, Department of Internal Medicine, VCU School of Medicine, Richmond, VA, United States; ^2^Department of Biomedical Engineering, Virginia Commonwealth University, Richmond, VA, United States

**Keywords:** cardiac MRI, anthracycline, cardiotoxicity, cardiac assessment, cancer treatment

## Abstract

The objective of this review article is to discuss how cardiovascular magnetic resonance (CMR) imaging measures left ventricular (LV) function, characterizes tissue, and identifies myocardial fibrosis in patients receiving anthracycline-based chemotherapy (Anth-bC). Specifically, CMR can measure LV ejection fraction (EF), volumes at end-diastole (LVEDV), and end-systole (LVESV), LV strain, and LV mass. Tissue characterization is accomplished through T1/T2-mapping, late gadolinium enhancement (LGE), and CMR perfusion imaging. Despite CMR’s accuracy and efficiency in collecting data about the myocardium, there are challenges that persist while monitoring a cardio-oncology patient undergoing Anth-bC, such as the presence of other cardiovascular risk factors and utility controversies. Furthermore, CMR can be a useful adjunct during cardiopulmonary exercise testing to pinpoint cardiovascular mediated exercise limitations, as well as to assess myocardial microcirculatory damage in patients undergoing Anth-bC.

## Introduction

Cardiovascular magnetic resonance (CMR) is a non-invasive imaging method that produces 3-dimensional images without using ionizing radiation and may accurately assess left ventricular (LV) myocardial structure, function (strain, volumes, ejection fraction), perfusion, and tissue characteristics across a variety of disease processes including heart failure, ischemic heart disease, non-ischemic cardiomyopathy, myocarditis, pericardial disease, and congenital heart disease (1).

Recently, CMR has been used to assess LV myocardial injury and identify cardiotoxicity in patients receiving anthracycline-based chemotherapy (Anth-bC) for the treatment of various cancers including breast, leukemia, lymphoma, bladder, and ovarian cancer (2). Cardiotoxicity is a condition defined as change in LV performance which can be assessed through CMR and is most commonly identified as a left ventricular ejection fraction (LVEF) absolute decline of ≥ 10%, or a decline to a value < 50% (3–6). CMR can determine LVEF and extracellular volume fraction (ECVF) and assess LV myocardial injury, helping to identify cardiotoxicity. Furthermore, CMR tissue-based characterization, such as late gadolinium enhancement (LGE) imaging, detects myocardial fibrosis, which can be indicative of LV injury, and T1/T2 mapping with CMR allows for accurate spatial visualization while assessing for cardiotoxicity.

This article will review the utilization of CMR for identifying cardiotoxicity in patients receiving Anth-bC, discuss recent clinical data and parameters that can be detected *via* CMR, address challenges, and finally, consider recent breakthroughs and new frontiers of research.

### Anthracyclines and cardiotoxicity

Anthracyclines are a class of anti-tumor agents including daunorubicin, doxorubicin, epirubicin, idarubicin, and valrubicin (2). Anthracyclines are commonly used as components of curative therapy for various types of cancers (2). For example, adjuvant chemotherapy containing anthracyclines decrease the 10-year risk of breast cancer recurrence from 47.4 to 39.4% when compared to breast cancer patients who received no chemotherapy (7), and increase overall survival among patients with Non-Hodgkin’s lymphoma (112 months, on average) when compared to lymphoma cancer patients who received a non-anthracycline regimen (94 months, on average; *p* = 0.0004) (8).

Although effective in eradicating tumors in breast, lymphoma, and soft tissue sarcoma cancers, anthracycline-related treatments may have a negative impact on LV function and promote heart failure (HF) (7, 9, 10). The mechanism by which anthracycline-induced LV myocardial injury occurs is multi-factorial and includes (a) inhibition of topoisomerase (Top) 2β, (b) generation of reactive oxygen species (ROS) (11), (c) downregulation of adiponectin (12), (d) promoting LV myocardial fibrosis (13), (e) promoting mitochondrial toxicity (14), and (f) promoting microvascular disease (15).

### What is cardiac magnetic resonance imaging and how can it assess anthracycline-induced cardiotoxicity?

Mechanistically, CMRs are based on the detection of signals from hydrogen nuclei (1). When a patient enters a scanner, hydrogen nuclei align with, and “precess” about the axis of the magnetic field and small magnetic pulses are delivered. Cine images used to evaluate cardiac volumes are acquired using steady state free precession (SSFP) pulse sequences, which allows for signals to be received and processed to produce an image of the spatial distribution of the spins of protons within the body (16). CMR is unique in its use of various imaging sequences: cine imaging determines morphology, T2-weighted imaging describes myocardial edema, perfusion characterizes ischemia, and LGE is used for scar and tagging for myocardial strain imaging (17). While CMR imaging techniques can identify cardiac structure, measure cardiovascular function, and characterize tissue, these techniques do not use ionizing radiation, which is particularly advantageous for use in oncology patients.

### Measuring left ventricular ejection fraction with cardiovascular magnetic resonance

**FIGURE 1:**
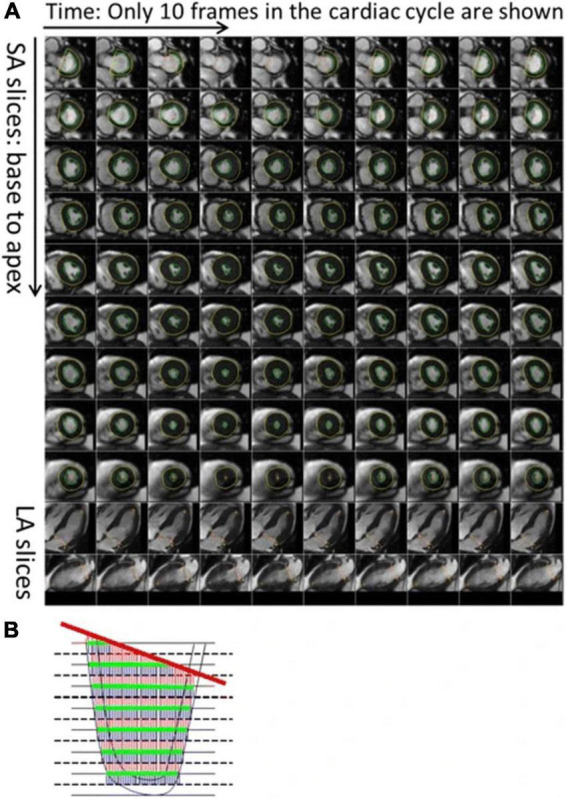
Description of the automatic process to calculate left ventricular (LV) volume and strain. As shown, **(A)** the LV endocardium and epicardium are automatically contoured on the LV short axis slices. The mitral valve base plane is detected on the long axis slices of the left ventricle and projected onto the LV short axis slices. **(B)** To calculate the LV volumes, slices are stacked and contoured areas are summed up. At the same time, the mitral valve base plane is intersected with the stack of slices to remove portions of the volume that are above the base plane and inside the left atrium. From (38) with permission.

Two common techniques to determine LVEF with CMR are the Simpson’s rule technique and the area-length technique (18). The Simpson’s rule technique (19, Figure 1) uses multiple short axial slices from the base to the apex of the myocardium. Next, the endocardial border is identified, and each intraventricular area is multiplied by slice thickness to determine LV volumes which are summed to calculate total LV volume without requiring assumptions about LV shape. Therefore, this method is especially useful among patients who exhibit cardiomyopathy or similar abnormalities of the myocardium. In situations in which the left ventricle assumes the shape of a prolate ellipsoid, the area-length technique may be used, as it encompasses only two slice acquisitions in LV apical views. Once LV volumes are determined, LVEF is calculated with the following formula: ([left ventricular end-diastolic volume (LVEDV)—left ventricular end-systolic volume (LVESV)]/LVEDV) *100. LVESV is the LV volume at the end of systole, with an average indexed value of 26 ± 6 mL/m^2^ for males and 24 ±5 mL/m^2^ for females, whereas LVEDV is the LV volume at the end of diastole, with an average indexed value of 81 ± 12 mL/m^2^ for males and 76 ± 10 mL/m^2^ for females (19).

In 53 men and women with breast cancer, leukemia, or lymphoma, LVEF decreased within 6 months after low to moderate doses of Anth-bC (10) (Table 1). Moreover, Ferreira de Souza et al. (20) used CMR to determine that among 27 women with breast cancer, the average LVEF declined by 12 absolute LVEF units (*p* < 0.001) at 351–700 days after anthracycline therapy of 240 mg/m^2^ (20). Additionally, the average LV mass index decreased by 19 g/m^2^, from 51.4 ± 8.0 to 36 ± 6 g/m^2^ (*p* < 0.001), and cardiac troponin T significantly increased (*p* < 0.001). Therefore, based on these studies, patients being treated with Anth-bC obtained CMRs before, 6, 12, and/or 24 months after anthracycline treatment.

**TABLE 1:** Summarizes the number of cancer patients, cancer type, chemotherapy, techniques to measure LVEF and/or LV mass, and the outcome of each study cited in this manuscript.

References	Number of cancer patients (male/female)	Cancer type	Chemotherapy	Techniques to measure LVEF and/or LV Mass	Outcome
Drafts et al. 10	53 (31/22)	Breast cancer, leukemia, or lymphoma	50–375 mg/m^2^ of doxorubicin equivalent chemotherapy	CMR: cine white blood steady state free precession techniques with 256×128 matrix; 40 cm field of view; 10-ms repetition time; 4-ms echo time; 20-degree flip angle; 8 mm thick slice; 40-ms temporal resolution	LVEF decline from 58 ± 1 to 53 ± 1% in 6 months (*p* = 0.0002)
Ferreira de et al. 20	27 (0/27)	Breast cancer	Anthracycline therapy of 240 mg/m^2^	CMR: cine imaging (steady-state free precession with TR 3.4 ms, echo time 1.2 ms, and in-plane spatial resolution 1.5 mm)	Mean LVEF decline by 12% to 58 ± 6% (*p* < 0.001) at 351–700 days after anthracycline therapy LV mass index decreased by 19 g/m^2^, from 51.4 ± 8.0 g/m^2^ to 36 ± 6 g/m^2^ (*p* < 0.001)
Melendez et al. 21	112 (78/34)	Breast cancer, Leukemia, Lymphoma, Renal Cell, or Sarcoma	Mixture of anthracyclines (72%), antimicrotubule agents (60%), alkylating agents (74%), and tyrosine-kinase inhibitors (51%)	CMR: cine white blood steady-state free precession techniques with a 256 × 128 matrix; 40-cm field of view; 10-ms repetition time; 4-ms echo time; 20-degree flip angle; slice 8-mm thick; 40-ms temporal resolution	26 patients developed significant declines in LVEF of >10% or to values <50% at 3 months Participants who dropped their LVEF due to decreases in LVEDV lost more LV mass than those who dropped their LVEF due to an increase in LVESV (*p* = 0.03)
Jordan et al. 31	61 (19/42)	Breast cancer, hematologic malignancies, or sarcomas	Average cumulative doxorubicin dose equivalent of 232 ± 103 mg/m^2^	CMR: cine short-axis white-blood steady-state free precession images were acquired encompassing the LV in 8-mm thick planes separated by 2-mm gaps; 40-cm field of view, 192 × 109 matrix, 10-ms repetition time, 1.12-ms echo time, 20° flip angle, 930 Hz/pixel bandwidth, and 40-ms temporal resolution	5% decline in both LVEF (*P*< 0.0001) and LV mass (*P* = 0.03)
Neilan et al. 30	91 (53/38)	Not reported	Anthracycline dose of 276 ± 82 mg/m^2^	CMR: successive short-axis cine images at end-diastole and systole LV mass by CMR was derived by the summation of discs method by multiplying myocardial muscle volume by 1.05 g/cm^3^	Inverse association between anthracycline dose and indexed LV mass (*r* = –0.67, *p* < 0.001) Indexed LV mass had the strongest association with MACE (HR = 0.89, chi-squared = 26, *p* < 0.001)
Sawaya et al. 36	43 (0/43)	Breast cancer	Doxorubicin (240 mg/m^2^), Epirubicin (300 mg/m^2^)	Transthoracic echocardiography using the Vivid 7 or E9 LVEF calculated from apical 4- and 2-chamber views using a modified Simpson’s biplane method	LVEF change from 0.65 ± 0.06 at baseline to 0.63 ± 0.06 at 3 months, 0.59 ± 0.05 at 6 months (*p* < 0.0001) Circumferential strain (%) from 18 ± 4 at baseline to 15 ± 4 at 3 months, 14 ± 3 at 6 months (*p* = 0.001)
Jolly et al. 38	72 (24/48)	Breast cancer (39%), lymphoma (49%), or sarcoma (12%)	Anthracycline (68%), Antimicrotubular agents (67%), Alkylating agents (78%), Tyrosine-kinase inhibitors (39%), Antimetabolites (6%)	Cine balanced steady state free precession (bSSFP) imaging was performed using breath-hold retrospective ECG gating to acquire a stack of short axis slices as well as 2 long axis views (2-chamber and 4-chamber)	The LVEF declined from 65 ± 7% at baseline to 62 ± 7% at 3 months (*p* = 0.0002). LV Strain changed from –18.81 ± 2.89 at baseline to –17.58 ± 3.08 at 3 months (*p* = 0.001) The correlation between LV strain from cine imaging and LVEF was *r* = –0.61 (*p* < 0.0001), and the 3-month changes in each measurement also correlated (*r* = –0.49, *p* < 0.0001)
Higgins et al. 42	20 (15/5)	Lung cancer (30%), renal cell carcinoma (25%), melanoma (15%), other (30%)	Nivolumab (50%), pembrolizumab (40%), ipilimumab (30%)	CMR: Steady state free precession (SSFP) cine imaging (repetition time = 3 ms, echo time = 1.5 ms, flip angle = 60°, 30 cardiac phases, 1.4 × 1.4 × 8 mm^3^ resolution) with retrospective ECG gating was acquired in the two-chamber, three-chamber, and four-chamber views, and in contiguous short axis slices of the left ventricle	LV strain was negatively correlated with LVEF (*r*_*s*_ = –0.64, *p* < 0.002)
de Barros et al. 47	112 (1/111)	Breast cancer	Doxorubicin and Cyclophosphamide, Paclitaxel, Trastuzumab	Transthoracic echocardiogram, including longitudinal strain assessment with 2D speckle-tracking echocardiography	LVWMA (OR = 6.25 [CI 95%: 1.03; 37.95], *p* < 0,05), LV systolic dimension (1.34 [CI 95%: 1.01; 1.79], *p* < 0,05) and global longitudinal strain by speckle tracking (1.48 [CI 95%: 1.02; 2.12], *p* < 0,05) were strongly associated with cardiotoxicity
Tahir et al. 49	39 (0/39)	Breast Cancer	Epirubicin-based chemotherapy	CMR: T2 mapping was performed using a free-breathing navigator-gated black-blood prepared gradient and spin-echo (GraSE) hybrid sequence in three short-axis slices; T1 mapping performed using a 5 s (3 s) 3 s MOLLI sequence with parameters: voxel size 2 × 2 × 10 mm^3^, echo time=0.7 ms, time to repetition=2.3 ms, partial echo factor =0.8, flip angle =35°, SENSE factor =2, linear phase encoding.	T1/T2 myocardial relaxation times increased at therapy completion when compared to baseline, thus indicating myocardial injury, and when assessed with other CMR parameters (LVEF), this predicted anthracycline-induced cardiotoxicity [sensitivity (78%, 44–95%) and specificity (84%, 72–92%)]
Toro-Salazar et al. 51	46 (33/13)	Acute myelogenous leukemia (21.7%), osteosarcoma (13%), Hodgkin lymphoma (10.9%), Ewing sarcoma (10.9%)	Average total cumulative dose of anthracyclines of 328 mg/m^2^	CMR: standard multislice, multiphase cine imaging using a steady-state free-precession acquisition technique (fast imaging employing steady-state acquisition [FIESTA]) in the 2-chamber, 4-chamber, and contiguous short-axis planes T1 mapping using modified Look-Locker with saturation recovery sequence (MLLSR	Post contrast T1 values of cancer patients were significantly lower than control patients (458 ± 69 versus 487 ± 44 milliseconds; *P* = 0.01)
Modi et al. 54	298 (108/190)	Breast cancer (38.6%), lymphoma (95%), leukemia (55%), sarcoma (21%), or other (4%)	Anthracyclines or trastuzumab	Cine CMR images were acquired in short-axis (every 10mm to cover the entire LV from the mitral valve plane through the apex) and three long-axis views using a steady-state free precession sequence LGE CMR was performed 10–15min after administration of gadolinium contrast (0.1–0.15mmol/kg), using 2D segmented inversion-recovery gradient-echo sequence in identical views as cine CMR	31 patients (10.4%) had LGE that ranged from 3.9–34.7% in extent, and an ischemic pattern was present in 20 (64.5%) of the 31 patients, yet these were no different in age-matched control patients
Jordan et al. 56	37 (8/29)	Breast cancer (57%), hematologic (43%)	Anthracyclines	CMR: Cine imaging parameters included a 360–400 mm field of view collected with a 256 × 160 matrix, a 20° flip angle, a 6 mm slice thickness with 4 mm slice gap, a 3–5 ms echo time, and an 8–10 ms repetition time T2 mapping: 360 × 360 field of view, 192 × 60 matrix, 70° flip angle, 6 mm slice thickness, acceleration factor of 2, T2 preparation pulses of 0, 24, 55 ms T1 mapping: Look-Locker inversion recovery sequence in mid-cavity short-axis slice pre-contrast and again at 12 and 25 min after contrast administration in contrast eligible participants	T1 and ECVF remain elevated 3 years after anthracycline-based treatment, independent of cardiovascular comorbidities or underlying cancer

**FIGURE 2:**
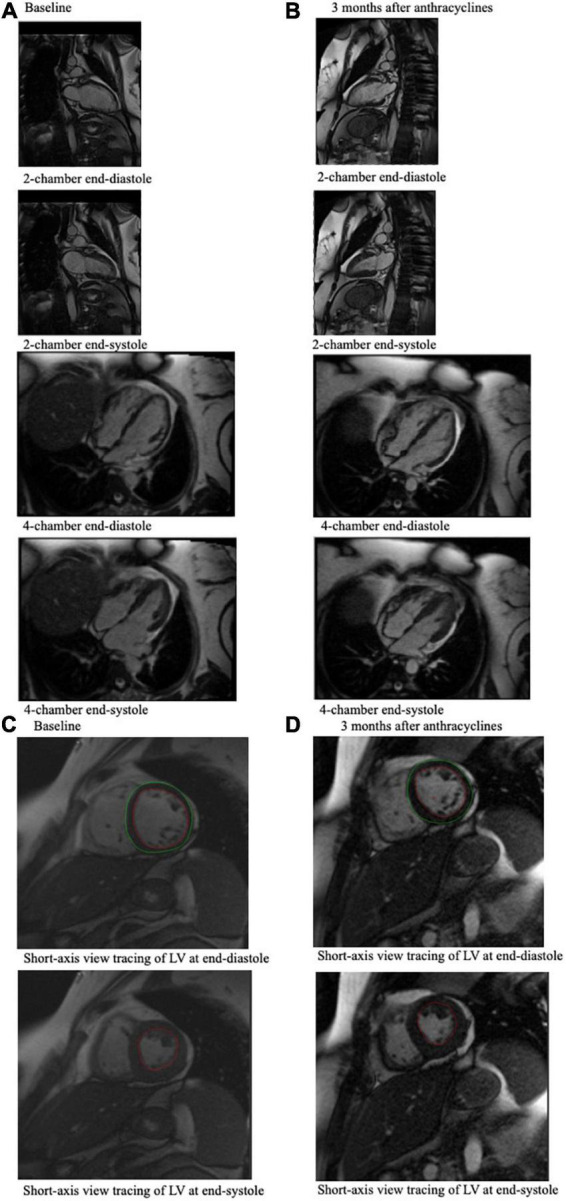
CMR end-diastole and end-systole frames from a cine loop for a 49-year-old breast cancer patient who experienced an associated LVEF decline from 61% at baseline **(A)** to 43% 3 months after anthracycline-based chemotherapy **(B)**, which was measured using these CMR images. **(A)** Shows 4-chamber and 2-chamber view CMR imaging of the cancer patient pre-chemotherapy, and **(B)** shows CMR imaging of the same cancer patient 3 months after chemotherapy. **(C)** Shows the short-axis view tracing of the LV at end-diastole and at end-systole at baseline, which can be compared to **(D)** which shows short-axis view tracings of the LV at end-diastole and end-systole of this patient at 3 months after chemotherapy. In these tracings, the red tracing outlines the blood pool in the left ventricle, and the green tracing outlines the left ventricle. These tracings, which indicate LVEDV and LVESV, are used to calculate LVEF.

Furthermore, a study by Melendez et al. (21) used CMR to determine that among 112 cancer patients (72% of whom received anthracyclines), 26 patients developed significant declines in LVEF of > 10% or to values < 50% at 3 months (21). Among these 26 patients, 19% were determined to have a decline in LVEF due to a decline in LVEDV, whereas 60% were determined to have a decline in LVEF due to an increase in LVESV. Systolic heart failure is associated with LVEF drops given by LVESV changes, but changes in LVEDV may be due to volume depletion, which is a common occurrence in cancer patients. Thus, the LVEF may decrease due to a decline in LVEDV or an increase in LVESV, and CMR allows for clinical determination of which case is present in a patient (Figure 2).

### Measuring left ventricular mass with cardiovascular magnetic resonance

In cancer patients, LV mass may decrease and result in greater end systolic wall stress, known as the Grinch Syndrome. Typically, indexed LV mass ranges from 50 to 86 g/m^2^ for males and 36–72 g/m^2^ for females (22) and can be determined by CMR, but in patients with the Grinch Syndrome, these values can decline and adversely affect the myocardium. The mechanism of cardiac atrophy involves suppression of pro-growth pathways, such as signaling events controlled by myocyte-enriched calcineurin-interacting protein-1 (hMCIP1) or Thioredoxin1 (Trx1), or direct stimulation of protein degradation (23–27).

**FIGURE 3:**
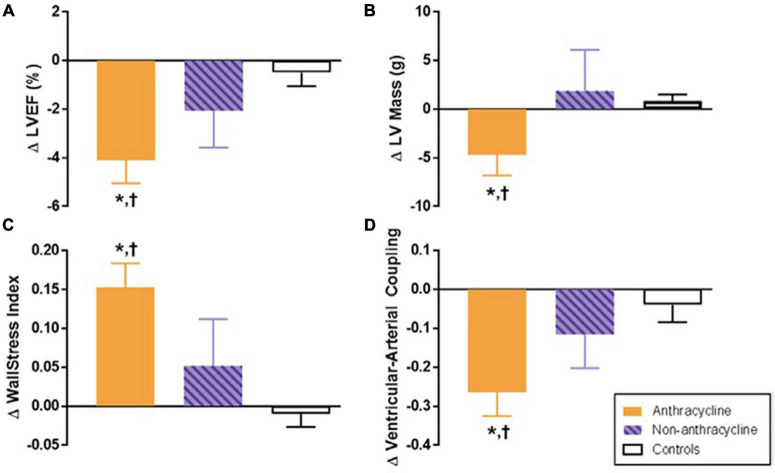
Six-month change in cardiovascular magnetic resonance (CMR)-derived left ventricular (LV) remodeling measurements after anthracycline-based chemotherapy. Six-month change in CMR-derived measurements of left ventricular remodeling in adults treated with anthracycline-based chemotherapy (Anth-bC, orange), non-Anth-bC (purple) for breast cancer or hematologic malignancy and cancer-free comparators of similar age (white). Compared with cancer-free comparators, those receiving Anth-bC had a significant decrease in LV ejection fraction (LVEF; **A** *P* < 0.01) and LV myocardial mass (**B**; *P* = 0.03) that occurred concurrently with increased end-systolic wall stress index (**C**; *P* < 0.01) and reduced ventricular-arterial coupling (**D**; *P* < 0.01). Changes among patients with cancer who received non–Anth-bC were not statistically different than those observed in non-cancer comparators (*P* > 0.15 for all). Data shown as mean ± SEM. *P < 0.05 for change from baseline. ^†^*P* < 0.05 vs. change in control. From Jordan et al. (31) with permission.

LV mass can be calculated by subtracting LVEDV from epicardial volume and multiplying by 1.05 g/mL (28). The Grinch Syndrome has been observed in patients receiving Anth-bC as early as 1 (29) and 6 months (31, Figure 3) after initiating treatment (30). Specifically, researchers conducted a cohort analysis with 61 cancer patients initiating Anth-bC, and assessed their cardiac function with CMR before and 6 months after initiating chemotherapy (31). The researchers observed a 5% decline in LV mass (*p* = 0.03) and LVEF (*p* < 0.0001) but did not observe this decline in patients receiving non-Anth-bC (*n* = 15) or control patients (*n* = 24). Additionally, patients receiving Anth-bC experienced a decline in Minnesota Living with Heart Failure Questionnaire scores over the 6-month period, which was associated with LV mass declines (*r* = –0.27; *p* < 0.01), yet not with LVEF declines (*r* = 0.11; *p* = 0.45), thus indicating that patients with declining LV mass while undergoing treatment with Anth-bC may experience worsening heart failure symptomatology that’s independent of LVEF (31).

Furthermore, in a study with 91 patients receiving Anth-bC, researchers found an inverse association between anthracycline dose and indexed LV mass (*r* = –0.67, *p* < 0.001; 32). The researchers also determined if patients experienced a major adverse cardiovascular event (MACE), which was defined as cardiovascular death, implantable cardioverter defibrillator therapy, and admission for heart failure (30). Among patients who experienced a MACE during the 27-month study period, indexed LV mass and glomerular filtration rate were lower while anthracycline dose was higher (30). Additionally, indexed LV mass had the strongest association with MACE (HR = 0.89, chi-squared = 26, *p* < 0.001) in a multivariable model (30). Therefore, determination of LV mass by CMR may be a predictor of adverse cardiovascular events (30).

However, LV mass has been shown to decrease in conditions of weightlessness, bed rest, and other situations of ventricular unloading. For example, LV mass index decreased by 15% after 12 weeks of bed rest in healthy individuals (32), and another study by de Groot et al. (33) reported a 25% decrease in LV mass in patients with spinal cord injury who did not exercise (27, 33). Therefore, both general status and Anth-bC may affect LV mass, so general status may be a confounding factor while observing LV mass decline in patients treated with Anth-bC.

### Measuring left ventricular strain with cardiovascular magnetic resonance

**FIGURE 4:**
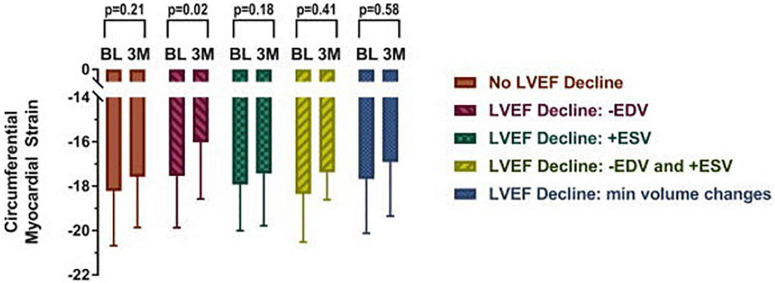
Mid-wall circumferential myocardial strain measured with CMR in cancer patients prior to (BL) and 3 months (3M) after initiation of chemotherapy categorized by underlying cause for left ventricular ejection fraction (LVEF) decline. Participants with a decline in LVEF due to a > 5 ml decrease in end diastolic volume exhibited subclinical deteriorations in myocardial strain. Myocardial strain changes were not observed in any other subgroup, including those with end systolic volume changes. From Jordan et al. (37) with permission.

LV strain is the percent change in length per unit of initial length (18). Two techniques are typically used to assess LV strain: Strain Encoding magnetic resonance imaging (SENC) and Displacement Encoding with Stimulated Echos (DENSE) (34). SENC imaging uses tagging of short-axis images with different z phases to calculate longitudinal strain, and DENSE imaging relies on tagging of short-axis images acquired with echoes to modulate pixels to their position in space. Both techniques have high spatial resolution and fast post-processing, but low temporal resolution. Both measure contraction in the longitudinal, circumferential, and radial directions (35). A study by Sawaya et al. (36) determined that an early decrease in longitudinal strain from baseline to 3 months among chemotherapy-treated breast cancer patients was an independent predictor of cardiotoxicity development at 6 months (36). As a decrease in LVEDV may lead to a decline in LV strain (37) (Figure 4), LV strain may also be affected by changes in LV volume. A study by Jolly et al. (38) determined that the correlation between LV strain from cine imaging and LVEF was *r* = –0.61 (*p* < 0.0001), and the 3-month changes in each measurement also correlated (*r* = –0.49, *p* < 0.0001) (38). Moreover, in a study by Houbois et al. (39), the authors suggest that feature-tracking strain can function as a confirmatory prognostic measure in addition to LVEF measurements when assessing for cardiotoxicity (39).

### Tissue tracking

LV strain can also be quantified and tracked with post-processing semi-automated methods, such as tissue tracking. Specifically, LV strain can be tracked over time with CMR feature tracking (CMR-FT), as CMR-FT follows myocardial boundaries and automates strain calculation. The tracked features are anatomic elements along the cavity-myocardial interface and throughout the cardiac cycle, and each feature is tracked by algorithms (40). Tissue tracking technology depends on a post-processing method known as optical flow, which recognizes features in an image to track and follow in a sequence of successive images (41). A comparable technique for image tracking is known as speckle tracking echocardiography (STE), which is used when the ventricular myocardium has a speckled appearance.

Tissue tracking methods initially identify a small window on an image, and the search for the most comparable image pattern in the subsequent frame (41). Any displacement that’s determined between the two tissue patterns is considered the local displacement (41). Typically, the minimum window dimension used in cardiology is 8 by 8 pixels, and it’s essential to optimize image quality, temporal resolution, and speed and magnitude of displacement while performing tissue tracking (41). Furthermore, circumferential strain has been shown to have better reproducibility in CMR-FT than STE, but STE produces more accurate results for longitudinal strain due to the high echogenicity of the fibrous annulus (41). A study by Higgins et al. (42) measured LV strain, *via* CMR-FT, and LVEF of 20 patients undergoing cardiotoxic chemotherapy, and among these patients, LV strain was negatively correlated with LVEF (*r*_*s*_ = –0.64, *p* < 0.002) (42).

The reproducibility and validity of CMR-FT have been assessed extensively (43). For example, CMR-FT was used to assess LV radial and circumferential strain in patients 5 years after undergoing Anth-bC, and it was found that circumferential strain was a reliable and reproducible measure of myocardial deformation, whereas radial strain measurement were unreliable (44).

### Measuring left ventricular wall motion with cardiovascular magnetic resonance

Because cine loops can be acquired during breath holding from nearly any tomographic plane, CMR also allows for visualization of LV wall motion (18). Moreover, LV wall thickening may be assessed using the center line method and myocardial tissue tagging. The center line technique creates a line down the center of the myocardium and draws evenly distributed perpendicular chords, where the length of each chord corresponds to the local wall thickness. However, when an imaging slice is not positioned perpendicular to the long axis of the left ventricle, the center line method may overestimate wall thickness. Tagging, on the other hand, can determine wall thickness across the LV myocardium by accounting for separation or intersections of tag lines.

Peak radial, longitudinal, and circumferential velocities are some of the parameters of LV wall motion (45). Typically, LV wall thickness is greater in males and younger patients, and systolic wall thickening (SWT) is highest in the lateral and apical myocardium, while wall motion (WM) is greatest in the lateral and basal myocardium (46). Additionally, if there is a wall motion abnormality in greater than two of seven parameters, then alterations to treatment progression may be recommended.

In a study by de Barros et al. (47), the LV wall motion of 112 cancer patients was analyzed to determine cardiotoxicity. Patients with LV segmental wall motion abnormalities (LVSWMA) were strongly associated with cardiotoxicity (47). Additionally, in a study by Jordan et al. (31), end-systolic wall stress index increased in cancer patients treated with anth-bC (*p* < 0.01; Figure 3) (31). LV wall motion abnormalities continue to be analyzed with CMR in cancer patients treated with anth-bC, and this remains an area of active study.

### Tissue characterization

**FIGURE 5:**
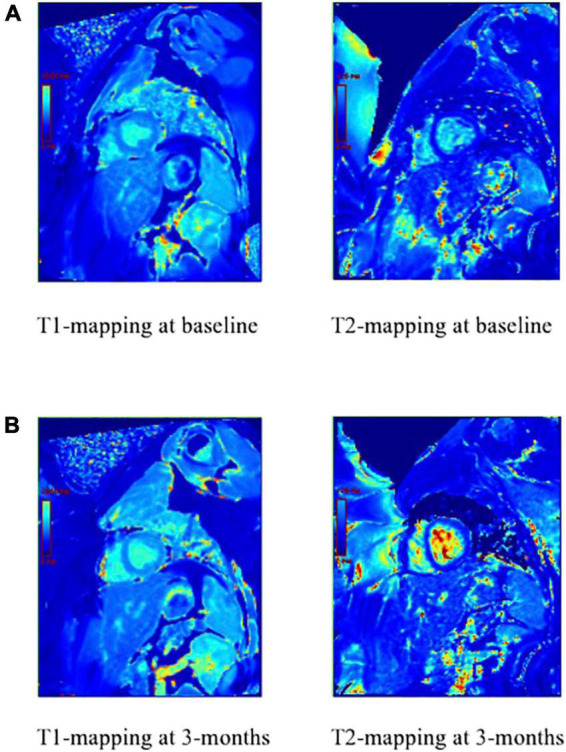
T1 and T2-mapping of the myocardium prior to anthracycline-based chemotherapy **(A)** and 3 months after anthracycline-based chemotherapy **(B)**. T1 has changed, which indicates active injury or ongoing fibrosis. T2 has also changed, which indicates increased water and active inflammation of the myocardium.

While CMR provides data regarding LV function, such as EF, strain, volume, and mass, it also provides tissue characterization data to assess myocardial fibrosis through methods that utilize gadolinium contrast and methods that do not use gadolinium contrast (native T1), such as T1 and T2 mapping. Longitudinal relaxation time (T1) and transverse relaxation time (T2) are properties determined by a tissue’s molecular composition (Figure 5). Thus, changes in T1 and T2 relaxation times, when compared to a healthy myocardium, may indicate cardiovascular injury such as edema and fibrosis. T1 and T2 measures have been validated by histological analysis to represent myocardial injury, interstitial fibrosis, inflammation, and edema of myocardial biopsy with anthracycline-induced cardiotoxicity (48). For example, a study by Tahir et al. (49) determined that among 39 women receiving chemotherapy, T1/T2 myocardial relaxation times increased, thus indicating myocardial injury, and when assessed with other CMR parameters, this predicted anthracycline-induced cardiotoxicity (49). Thus, T1/T2 mapping have clinical implications in potentially predicting cardiotoxicity following anthracycline treatment. Furthermore, Galan-Arriola et al. (50) found that in 20 pigs, T2 mapping during doxorubicin treatment detects intra-cardiomyocyte edema at 6 weeks, which is earlier than T1 mapping, LV motion, and extracellular volume quantification (50). A study by Toro-Salazar et al. (51) determined that among 46 long-term childhood cancer survivors who received cumulative anthracycline dose ≥ 200 mg/m^2^ and exhibited normal systolic function, post contrast T1 values were significantly lower than control patients (458 ± 69 vs. 487 ± 44 ms; *P* = 0.01) (51).

**FIGURE 6:**
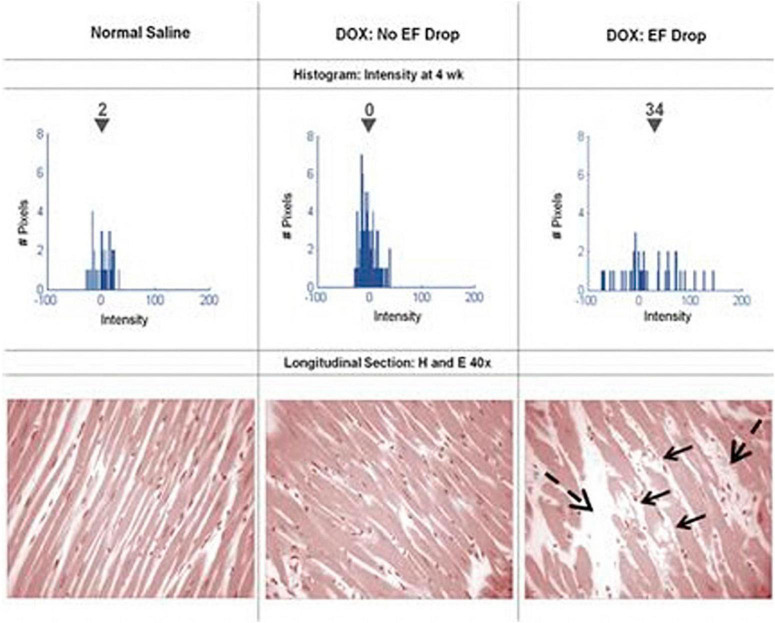
Serial histograms of myocardial LGE signal intensity (**top**, mean intensity shown above the *inverted black triangles*) and corresponding histopathology **(bottom)** of individual animals 4 weeks after receipt of normal saline **(left)**, doxorubicin without an LVEF drop **(middle)**, and doxorubicin with an LVEF drop **(right)**. Vacuolization (*arrows*) and increased extracellular space (*dashed arrows*) were observed in animals with doxorubicin cardiotoxicity. From Lightfoot et al. (53) with permission.

One CMR technique that assesses myocardial fibrosis using gadolinium contrast is LGE, and inversion recovery (IR) with magnitude reconstruction is the pulse sequence most widely used for LGE (52). Roughly 10 min after gadolinium contrast injection, it is evenly distributed, and fibrosis will show with high signal intensity due to the increase in stroma. Anthracycline use has been associated with LGE in some studies, yet the extent of this association is still being researched. For example, in an animal model of doxorubicin cardiotoxicity, greater LGE signal intensity in the myocardium was associated with future LV systolic dysfunction, vacuolization, and extracellular volume (53) (Figure 6). However, Modi et al. (54) determined that among 298 patients receiving anthracyclines or trastuzumab, 31 (10.4%) had LGE that ranged from 3.9 to 34.7% in extent, and an ischemic pattern was present in 20 (64.5%) of the 31 patients, yet these were no different in age-matched control patients (54).

**FIGURE 7:**
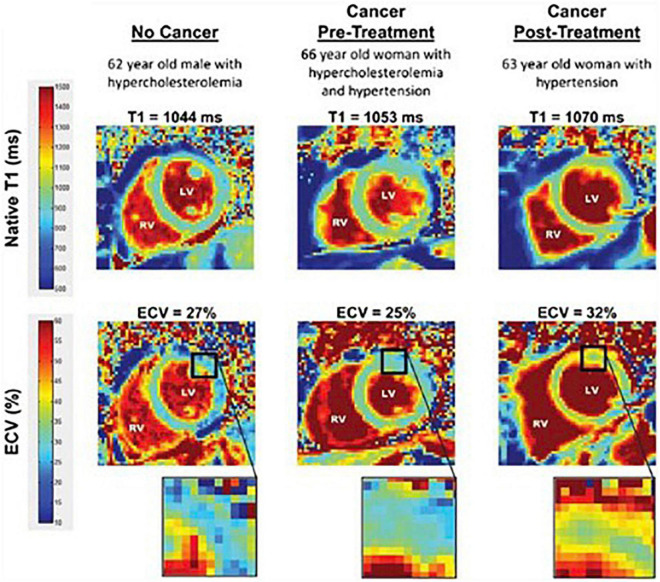
Comparison of T1 and ECV map images. Representative left ventricular (LV) short-axis native T1 (top row) and extracellular volume (ECV, bottom row) maps are shown in similarly aged participants. The LV and right ventricular (RV) blood pool cavities are noted. On each image, the color of pixels in the images (color scales on left) identifies the native T1 (milliseconds) and ECV (%). Insets on the ECV maps demonstrate the change in color intensity within the anterolateral wall of each ventricle. As shown, ECV is elevated in the cancer survivor previously treated with anthracycline-based chemotherapy. From Jordan et al. (56) with permission.

**FIGURE 8:**
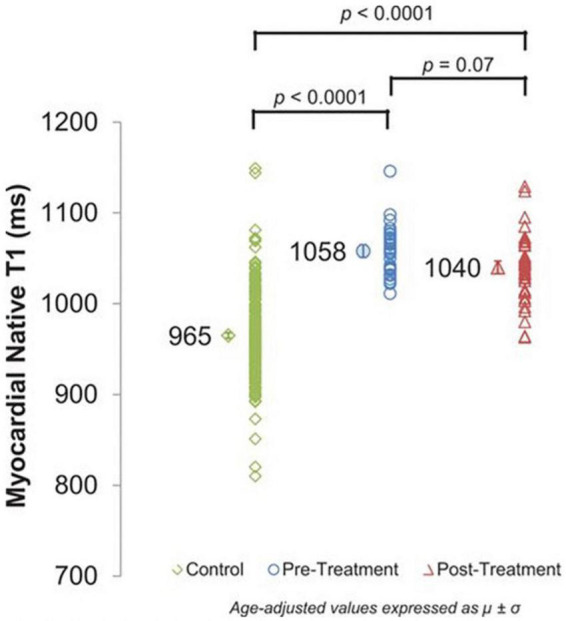
Cardiovascular magnetic resonance (CMR) assessments of myocardial T1. Myocardial native T1 of control participants without cancer (965 ± 3 ms) and cancer pretreatment participants (1,058 ± 7 ms) compared with cancer survivors treated with anthracycline chemotherapy (1,040 ± 7 ms). Myocardial T1 is elevated in both cancer groups, reflecting potential myocardial fibrosis compared with cancer-free control participants (*P* < 0.0001 for both). From Jordan et al. (56) with permission.

**FIGURE 9:**
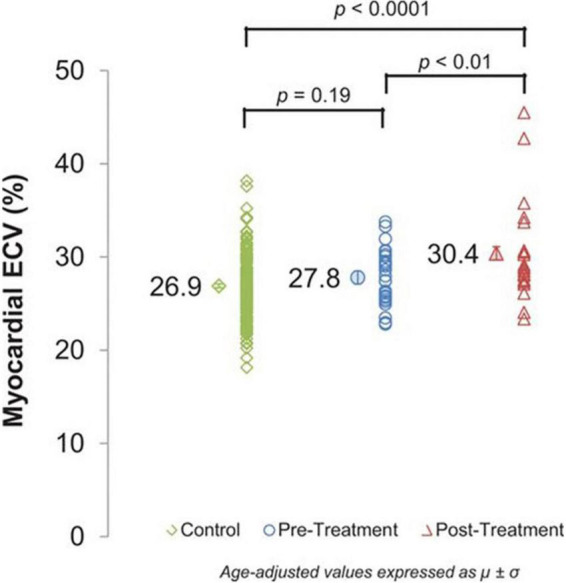
Cardiovascular magnetic resonance (CMR) assessments of myocardial extracellular volume (ECV). Myocardial ECV measured by cardiovascular magnetic resonance in controls (26.9 ± 0.2%) and cancer pretreatment participants (27.8 ± 0.7%) compared with cancer survivors treated with anthracycline chemotherapy (30.4 ± 0.7%). ECV incrementally increases across the groups (*P*< 0.0001) with a significant increase in post-treatment ECV values compared with pretreatment ECV values (*P*< 0.01). From Jordan et al. (56) with permission.

When T1-maps are collected both with and without contrast in a patient, the extracellular volume fraction (ECVF) can be calculated by first determining the partition coefficient from the slope of 1/T_*myocardium*_ vs. 1/T1_*blood*_, and then using the following formula: $ECVF=\left(1-hematocrit\right)\frac{\left(\frac{1}{T1_{myopost}}-\frac{1}{T1_{myopre}}\right)}{\left(\frac{1}{T1_{bloodpost}}-\frac{1}{T1_{bloodpre}}\right)}$ (55). A blinded CMR analysis of T1 and ECVF was conducted among 327 individuals (37 breast cancer patients, 54 cancer survivors, and 236 cancer-free patients) by Jordan et al. (56). Figure 7 (56) shows T1 images and ECVF measurements from a participant in each of the three study groups (no cancer, cancer pre-treatment, and cancer post-treatment). The researchers determined that T1 (56) (Figure 8) and ECVF (56) (Figure 9) remain elevated 3 years after anthracycline-based treatment, independent of cardiovascular comorbidities or underlying cancer, thus indicating that this T1 and ECVF elevation are related to receipt of anthracyclines.

CMR perfusion imaging can further characterize tissue (57). Perfusion defects can identify coronary artery disease (CAD), which shares many risk factors with cardiotoxicity (58, 59). Additionally, a retrospective study by Li et al. (60) determined that patients with cancer have higher risk of developing CAD when compared to non-cancer patients (OR: 2.024, 95% CI: 1.475–2.778, *p* < 0.001; 60). Perfusion imaging may also allow for assessment of myocardial perfusion reserve (MPR), which is the maximum increase in myocardial blood flow above baseline conditions, by quantifying myocardial blood flow at rest and during stress. Changes in MPR may elucidate the underlying pathophysiology of cardiomyopathies, such as coronary microvascular dysfunction and CAD, which increases risk of cardiotoxicity for patients undergoing potentially cardiotoxic chemotherapy.

### Clinical decision-making

CMR accurately assesses LV function and structure, and this allows for physicians to use findings in clinical decision-making. Cardiotoxicity is defined as a decline in LVEF by > 10% to a value < 50% while undergoing cancer treatment (59). Probable subclinical cardiotoxicity is defined as an LVEF decline by > 10% to a value ≥ 50% with a fall in global longitudinal strain (GLS) > 15% while undergoing cancer treatment (61). Possible subclinical cardiotoxicity is defined as an LVEF decline by < 10% to a value < 50% or a relative percentage reduction in GLS by > 15% from baseline while undergoing cancer treatment (61). If a patient falls into the cardiotoxicity, probably subclinical cardiotoxicity, or possible subclinical cardiotoxicity categories, as defined above, then a referral to cardio-oncology should be considered (61). However, if they do not fall into these categories, then treatment may continue as planned and surveillance imaging should be maintained (61). It’s important to note that clinical suggestions may vary depending upon consensus statements. Generally, in clinical practice, echocardiography remains the first line myocardial imaging technique due to access and affordability.

### Challenges

Although CMR is the gold standard for assessing myocardial function, challenges persist. CMR availability and portability are limited, which may contribute to less widespread use of CMR. A CMR scanner is 4–10 times the cost of a standard 3D echocardiography system (62). However, a retrospective study with 361 patients indicated that the use of CMR to assess myocardial function and structure saved roughly $2,308/patient due to avoidance of invasive procedures and further diagnostic testing (63). Additionally, CMR exams may take up to an hour longer than echocardiography exams. Thus, cost, accessibility, and time are all factors that may affect a patient’s decision to proceed with CMR imaging.

Feasibility to perform CMR on all patients must also be considered. Specifically, some patients require anxiolytics for claustrophobia while undergoing CMR, which may lower their heart rate and blood pressure during the exam, and thus affect findings. Additionally, patients with certain implants may not undergo magnetic resonance imaging, as artifacts can affect imaging outcome.

### New frontiers and next avenue of research

As CMR continues to serve as a robust and reliable tool for monitoring a patient’s progression through cardiotoxic treatment, as well as other myocardial injuries, the imaging modality continues to improve. Artificial intelligence (AI) and machine learning (ML) have become increasingly prominent in the field of CMR, improving the efficiency and accuracy of assessing cardiac function (64). A deep learning algorithm was developed to automatically segment right and left ventricular endocardium and epicardium to measure mass and function- a process that is often time-consuming and challenging to perfect (65–67). The algorithm was tested on various datasets, and it was found that measurements acquired by the algorithm were comparable to those of human experts in the field. Automating segmentation would save time and resources while assessing CMRs for cardiotoxic changes in cancer patients, and this would be especially useful in a clinical setting.

Moreover, the applications of CMR expand well beyond the field of cardio-oncology. Stress CMR images may be obtained to assess patients’ heart function under myocardial stress, which provides prognostic insight. Specifically, a retrospective cohort study by Antiochos et al. (68) obtained vasodilator stress perfusion CMRs in 1,698 patients without a history of coronary artery disease (CAD) (68). Stress CMR perfusion imaging reclassified 60.3% of patients who were in the intermediate pretest risk category, thus indicating that stress CMR imaging provides valuable information beyond clinical factors in risk stratifying patients at intermediate risk for cardiovascular death and non-fatal myocardial infarction (69). Additionally, CMR stress perfusion imaging, which allows for quantification of myocardial blood flow at rest and during stress, may help with identifying early vascular changes following Anth-bC (57). Myocardial perfusion defects have been detected 6–24 months post-radiation therapy in 40% of cancer patients using nuclear SPECT imaging, which were found to be associated with corresponding wall-motion abnormalities (70). Furthermore, Sioka et al. (71) conducted a multiple regression analysis which showed more abnormalities in myocardial perfusion imaging of left breast cancer patients who underwent radiation therapy when compared to controls (*p* = 0.0001); (71). Thus, analysis of CMR stress perfusion imaging may help refine clinical practice of radiation therapy in cancer patients.

**FIGURE 10:**
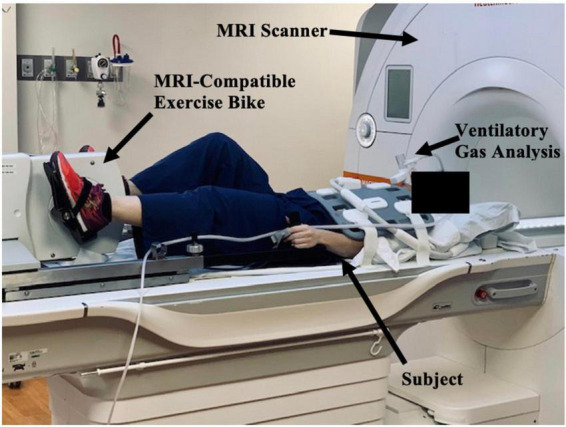
Study set-up with magnetic resonance imaging (MRI)-compatible exercise bike positioned in CMR imaging scanner. The subject, MRI scanner, MRI-compatible exercise bike, and ventilatory gas analysis are indicated by black arrows. The subject lies outside the MRI scanner and will pedal on an exercise bike as they move through the scanner. Ventilatory gas analysis is simultaneously performed while a cardiac MRI is obtained.

**FIGURE 11:**
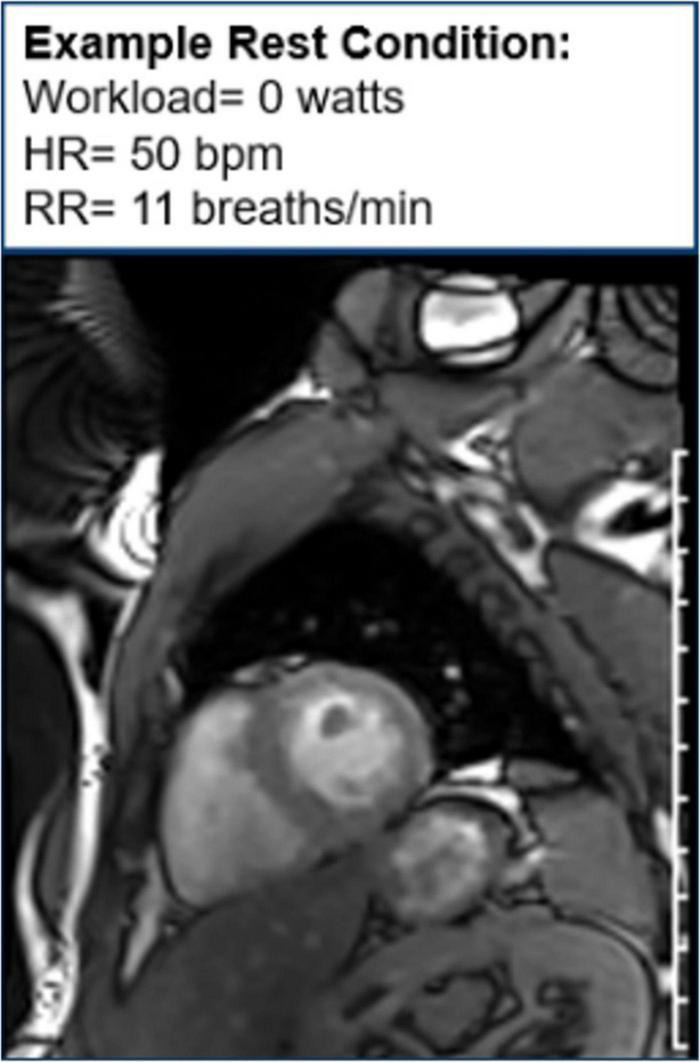
Cine image at rest. One image of a cine loop at the end short-axis view of the myocardium. The left ventricle is gray, while the blood pool is white. At rest, the workload is 0 w, and the patient’s heart rate is 50 bpm and respiratory rate is 11 breaths/min.

**FIGURE 12:**
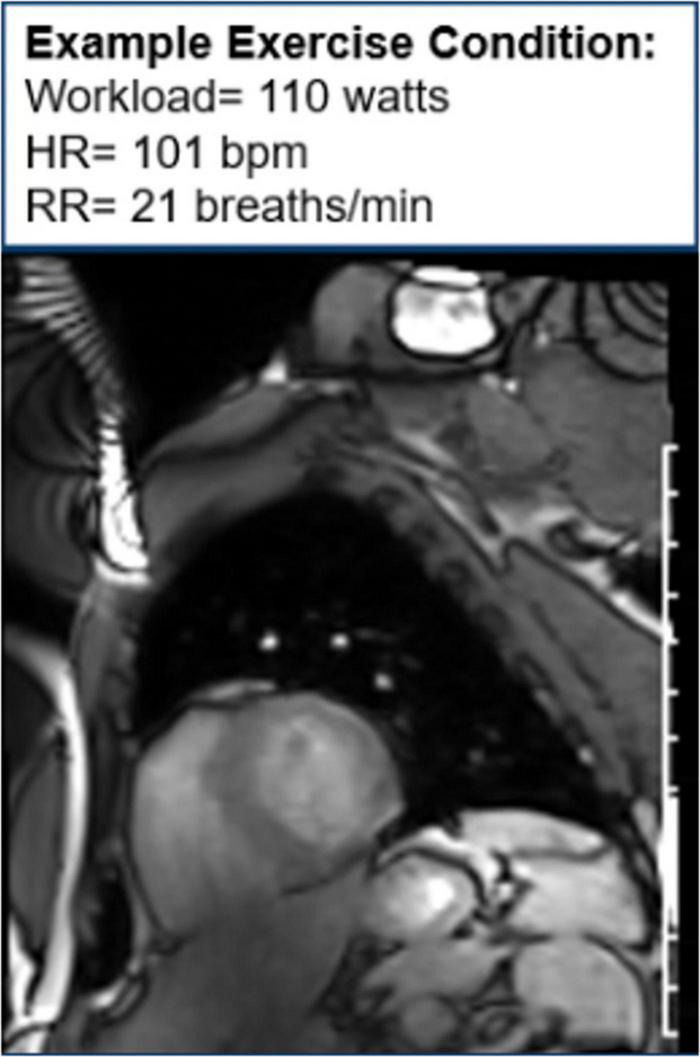
Cine image during exercise. One image of cine loop at the end short-axis view of the myocardium. The left ventricle is gray, while the blood pool is white. During exercise, the workload is 110 w, and the patient’s heart rate is 101 bpm and respiratory rate is 21 breaths/min.

Moreover, CMR can be used to assess cardiac function during exercise testing (72) and has even been utilized in hematologic cancer survivors by placing an exercise bike in the magnetic resonance imaging (MRI) scanner (Figure 10). Cine images at rest and during exercise are depicted (Figures 11, 12), as this technology allows for myocardial function to be compared at baseline and during exercise (73).

**FIGURE 13:**
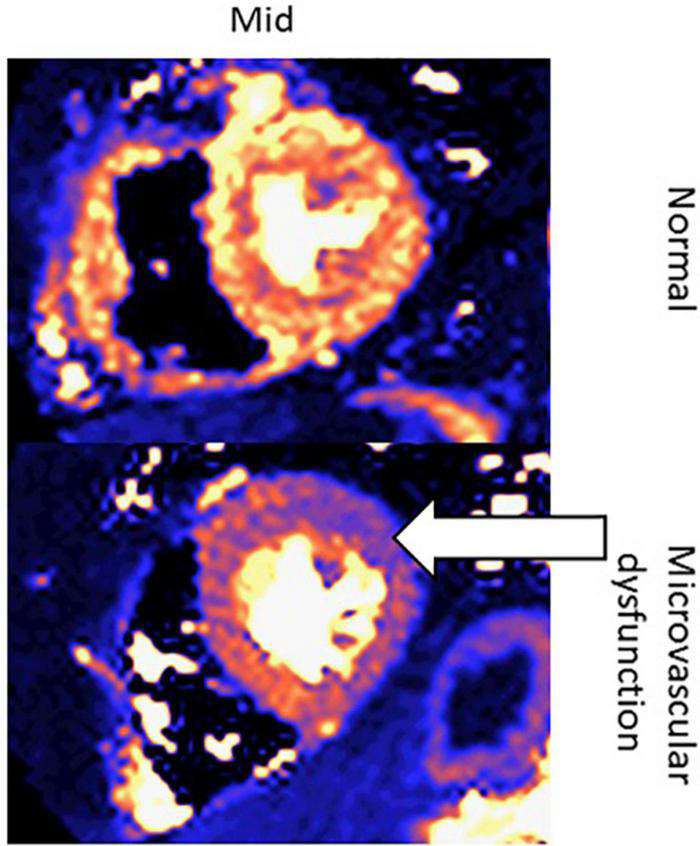
Quantitative perfusion CMR assessment of myocardial microcirculatory damage in a normal patient vs. cancer patient undergoing cardiotoxic chemotherapy. These are two left ventricular short-axis images of the mid-segment myocardium. The top row contains an image from a healthy patient, whereas the bottom row contains an image from a patient with microvascular dysfunction due to chemotherapy. The white arrow indicates the area of the left ventricle with the greatest microvascular dysfunction.

Another new application of CMR in oncology patients may be to evaluate microcirculatory damage, as one of the effects of cardiotoxic chemotherapy is microcirculatory damage to the myocardium (74). Studies have shown that cardiotoxic chemotherapies lead to a decrease in nitric oxide-mediated dilation in endothelial cells, which is associated with increased risk of various cardiovascular risk factors, such as heart failure and hypertension (75). One study assessing the direct effect of doxorubicin on human coronary microvascular function *ex vivo* found that among adult human coronary microvessels treated with doxorubicin, flow-mediated dilation (FMD) and coronary arteriolar function were significantly impaired (76). Moreover, CMR has been used to assess microcirculatory damage, as seen in Figure 13 (76–79).

With the development of real-time (RT) CMR, advances are also being made in CMR efficiency. By allowing for high spatial and temporal resolution during free breathing and without ECG synchronization, RT CMR reduces the total exam time from 12 to 15 min for SSFP to evaluate LV function in under 2 min. Although there have not been many studies assessing the accuracy of SSFP vs. RT CMR among cancer patients, the RT CMR technique may be useful in improving efficient resource utilization.

## Discussion and conclusion

As a non-invasive cardiac imaging technique, CMR assesses LV function, tissue characterization, and myocardial injury. LV function is accurately assessed through determination of LVEF, volumes at end-diastole (LVEDV), and end-systole (LVESV), strain, mass, and wall motion. Various techniques may be used for tissue characterization, some of which use gadolinium contrast (LGE and T1-mapping), and some of which are non-contrast methods (T1/T2 mapping). Significant clinical changes in LV function may correlate with cardiotoxicity, and CMR is especially useful in monitoring cardiac function throughout treatment with potentially cardiotoxic chemotherapy.

When compared to other modalities, CMR provides a variety of information while being non-ionizing, non-invasive, and accurate, thus making it advantageous for routine cardiac monitoring among oncology patients in a clinical setting. Additionally, CMR assesses LV volumes and can characterize tissue to assess myocardial fibrosis. Challenges persist, however, while using CMR in cancer patients, such as cost, efficiency, and accessibility. As new frontiers of research with CMR are constantly discovered, its applications and techniques continue to expand and improve, respectively.

## Author contributions

LM drafted the initial manuscript. WH, WB, and JJ critically reviewed the manuscript. All authors approved the final manuscript.
